# Editorial: p53 in cancer therapy: the impact of mutations on the genome guardian

**DOI:** 10.3389/fimmu.2026.1838954

**Published:** 2026-04-13

**Authors:** Manzoor A. Mir, Aijaz Bhat, Muzafar Ahmad Macha, Umar Mehraj, Nandini Verma, Gowhar Masoodi

**Affiliations:** 1 Cancer Biology Lab, Department of Bioresources, School of Biological Sciences, University of Kashmir, Srinagar, India; 2 Sidra Medicine, Doha, Qatar; 3 Watson-Crick Centre for Molecular Medicine, Islamic University of Science and Technology, Awantipora, Kashmir, India; 4 Duke University, Durham, NC, United States; 5 Advanced Centre for Treatment, Research and Education in Cancer (ACTREC), Mumbai, India

**Keywords:** biomarkers, co-mutations, gain-of-function (GOF), immune evasion, immunotherapy, p53, precision oncology, therapy resistance

The tumor suppressor p53, known as the “guardian of the genome,” is crucial for maintaining cellular homeostasis by regulating DNA repair, apoptosis, senescence, and metabolic equilibrium. Modifications in the TP53 gene occur most frequently in human cancers, substantially transforming tumor biology. In addition to mere loss-of-function (LOF), mutant p53 proteins frequently attain gain-of-function (GOF) characteristics that actively promote tumor development, treatment resistance, and immune evasion. This Research Topic, “p53 in Cancer Therapy: The Impact of Mutations on the Genome Guardian,” assembles a series of research that collectively underscore the diverse effects of TP53 mutations across various cancer types, highlighting their translational and therapeutic significance.

Shen et al. presented a thorough review on the role of TP53 in osteosarcoma, highlighting the contributions of both loss-of-function (LOF) and gain-of-function (GOF) mutations to genomic instability, metabolic reprogramming, and immune evasion. Their research emphasizes that mutant p53 not only interferes with traditional tumor suppressive pathways but also actively enhances oncogenic signaling, confounding therapeutic targeted approaches. The authors additionally underscore novel strategies, such as targeting metabolic vulnerabilities and non-coding RNA networks, as possible pathways to surmount TP53-driven resistance. Shi et al. demonstrate the significance of TP53 mutations on the tumor microenvironment and the development of therapy resistance by investigating an EGFR/TP53 co-mutated non-small cell lung cancer (NSCLC). This study utilized single-cell transcriptomics to identify multiple tumor cell groups that collaboratively create an immunosuppressive microenvironment. Significantly, signaling pathways including MDK, MIF, and TGF-β were identified as promoters of CD8+ T-cell exhaustion, whereas cancer-associated fibroblasts helped in the recruitment of regulatory T-cells, thereby facilitating immune evasion and resistance to targeted therapy. This work underscores the need of evaluating TP53 mutations with other oncogenic changes, as their synergistic effects can substantially affect therapy outcomes. Tornesello et al. investigated the prognostic implications of simultaneous TP53 and TERT promoter mutations in bladder cancer, deepening the understanding of co-mutations. Their findings indicate that patients displaying both alterations show markedly reduced overall survival, along with increased tumor mutational burden and microsatellite instability. These findings underscore that TP53 mutations never function independently; rather, their interplay with other genetic alterations enhances tumor heterogeneity and clinical severity. Song et al. further substantiate this concept by identifying LIG1 as a novel biomarker in bladder cancer. Their research demonstrated that LIG1 is linked to critical oncogenic mechanisms, including as DNA replication and the p53 signaling pathway, and contributes to immune cell infiltration in the tumor microenvironment. Collectively, these investigations underscore the complex network of p53-related pathways and their potential utility as diagnostic and prognostic instruments. Sun et al. devised a novel nomogram for predicting cervical cancer risk by combining p53 genotype, high-risk HPV infection status, and hematological markers. Their model exhibited significant predictive accuracy, highlighting the potential of integrating genetic and clinical information to enhance early identification and risk stratification. The interplay between p53 polymorphisms and HPV infection underscores the intersection of viral oncogenesis with host genetic variables, hence confounding the function of p53 in cancer progression. The review by Savostyanova et al. offers a thorough evaluation of p53-based immunotherapy approaches from a therapeutic perspective. The authors examine the prospect of targeting p53-derived neoantigens, especially those resulting from hotspot mutations, as viable candidates for adoptive T-cell treatments and cancer vaccines. Regardless promising early-phase outcomes, challenges such as restricted response duration and tumor immune evasion persist as substantial impediments. The findings correspond closely with the data of Shi et al., indicating that mutant p53-induced immunosuppressive microenvironments limit efficient immune responses, so proposing that successful therapeutic options must concurrently address both tumor-intrinsic and microenvironmental aspects.

In addition to these biological and therapeutic discoveries, Granata et al. emphasize the essential significance of imaging in assessing responses to cancer treatment. Their research underscores the necessity of incorporating molecular attributes, such as TP53 status, into radiological evaluation systems to enhance treatment oversight and decision-making. This multidisciplinary approach emphasizes the necessity for collaboration among molecular biologists, clinicians, and radiologists in the context of precision oncology.

The works under this Research Topic converge on numerous significant insights. TP53 mutations exhibit significant heterogeneity and are context-dependent, requiring tailored therapeutic strategies. Secondly, mutant p53 is crucial in influencing the tumor microenvironment, especially in facilitating immune evasion and resistance to therapy. The interaction between TP53 mutations and concurrent genetic changes markedly affects disease development and clinical outcomes. The incorporation of p53-associated indicators into clinical and computational frameworks has significant potential for enhancing cancer diagnosis and prognosis.

Future research should concentrate on elucidating the structural and functional diversity of TP53 mutations and their context-dependent impacts across various cancer types. Progress in single-cell sequencing, artificial intelligence, and multi-omics approaches will likely yield enhanced understanding of p53-regulated networks. Furthermore, the advancement of innovative therapeutic approaches focused on reinstating wild type p53 functionality or exploiting on the shortfalls of mutant p53 is an essential objective. Approaches like small-molecule reactivators, gene editing technologies, and p53-targeted immunotherapies have promising opportunities but necessitate additional validation in clinical environments.

This Research Topic offers a thorough examination of the advancing domain of p53 research in cancer treatment. The compiled papers emphasize the problems and potential related to addressing the genome guardian by integrating molecular mechanisms with therapeutic applications. Ongoing interdisciplinary collaboration will be crucial to convert these findings into viable therapeutic approaches and eventually enhance patient outcomes.

